# Supplementary material for: Emergence of cascading dynamics in interacting tipping elements of ecology and climate
Source: R Soc Open Sci. 2020 Jun 24;7(6):200599. doi: 10.1098/rsos.200599 (PMC7353982; doi:10.1098/rsos.200599)
Supplement: Additional results figures [file rsos200599supp1.pdf]

# Supplementary Material
# Emergence of cascading dynamics in interacting tipping elements of ecology and climate

Ann Kristin Klose*[1,2], Volker Karle[1,3], Ricarda Winkelmann[1,4], and Jonathan F. Donges†[1,5]

[1]Earth System Analysis, Potsdam Institute for Climate Impact Research, Member of the Leibniz Association, Telegrafenberg A31, 14473 Potsdam, Germany
[2]Carl von Ossietzky University Oldenburg, Oldenburg, Germany
[3]Institute of Science and Technology Austria, Am Campus 1, 3400 Klosterneuburg, Austria
[4]Department of Physics and Astronomy, University of Potsdam, 14469 Potsdam, Germany
[5]Stockholm Resilience Centre, Stockholm University, 10691 Stockholm, Sweden

June 12, 2020

*email: akklose@pik-potsdam.de
†email: donges@pik-potsdam.de

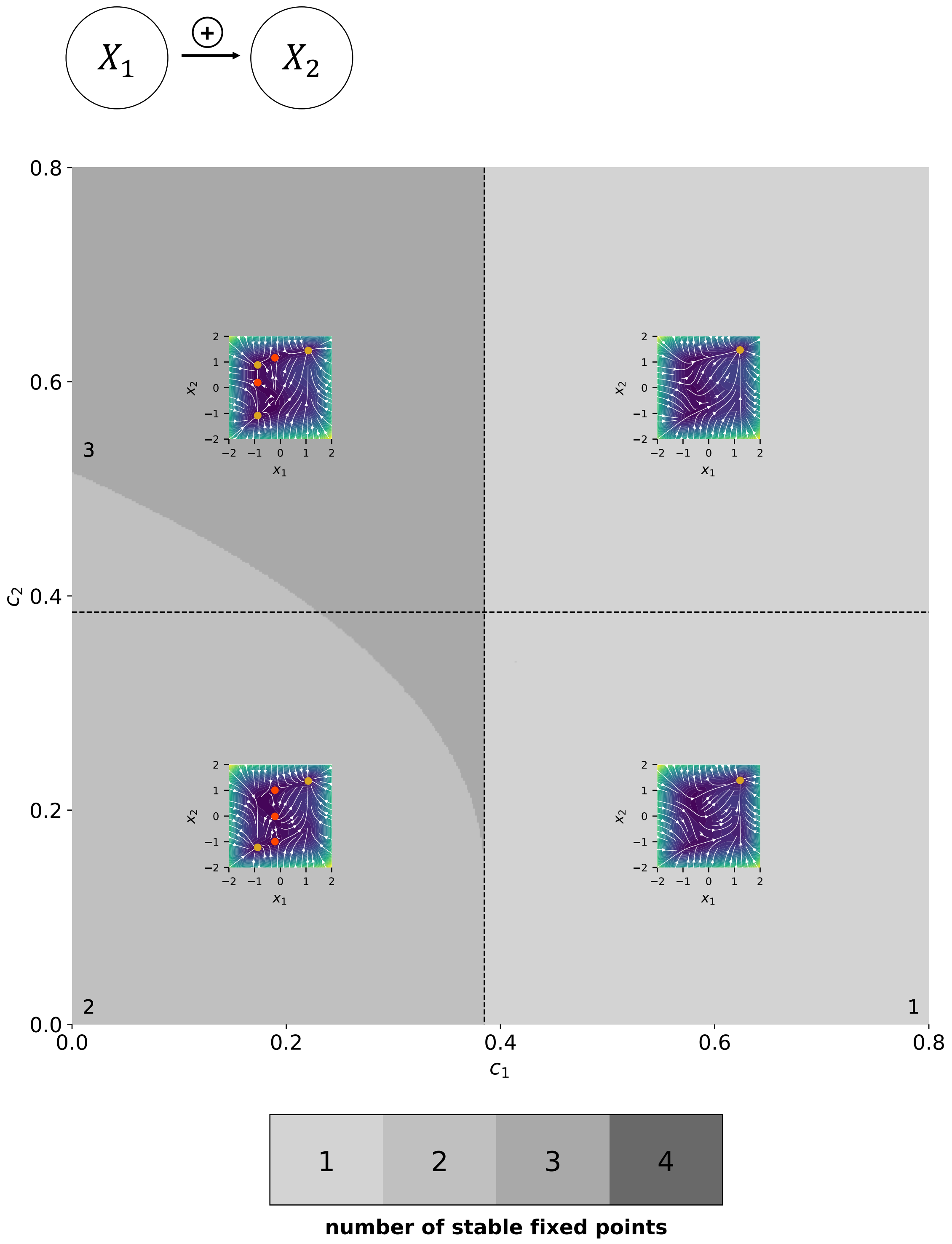


Figure 1: Number of stable fixed points and phase space portraits in a master–slave system with a high positive coupling strength $d_{12} = 0.9 > 0$ depending on the control parameters $c_1$ and $c_2$. The dashed lines represent the intrinsic tipping point of the respective subsystem. The phase space portraits allow to derive the possible critical transitions in the master–slave system. Within the phase space portraits stable fixed points are shown in orange, while unstable fixed points are shown in red. The background colour indicates the normalized speed $v = \sqrt{\dot{x}_1^2 + \dot{x}_2^2}/v_{max}$ going from close to zero (purple) to fast (yellow-green).

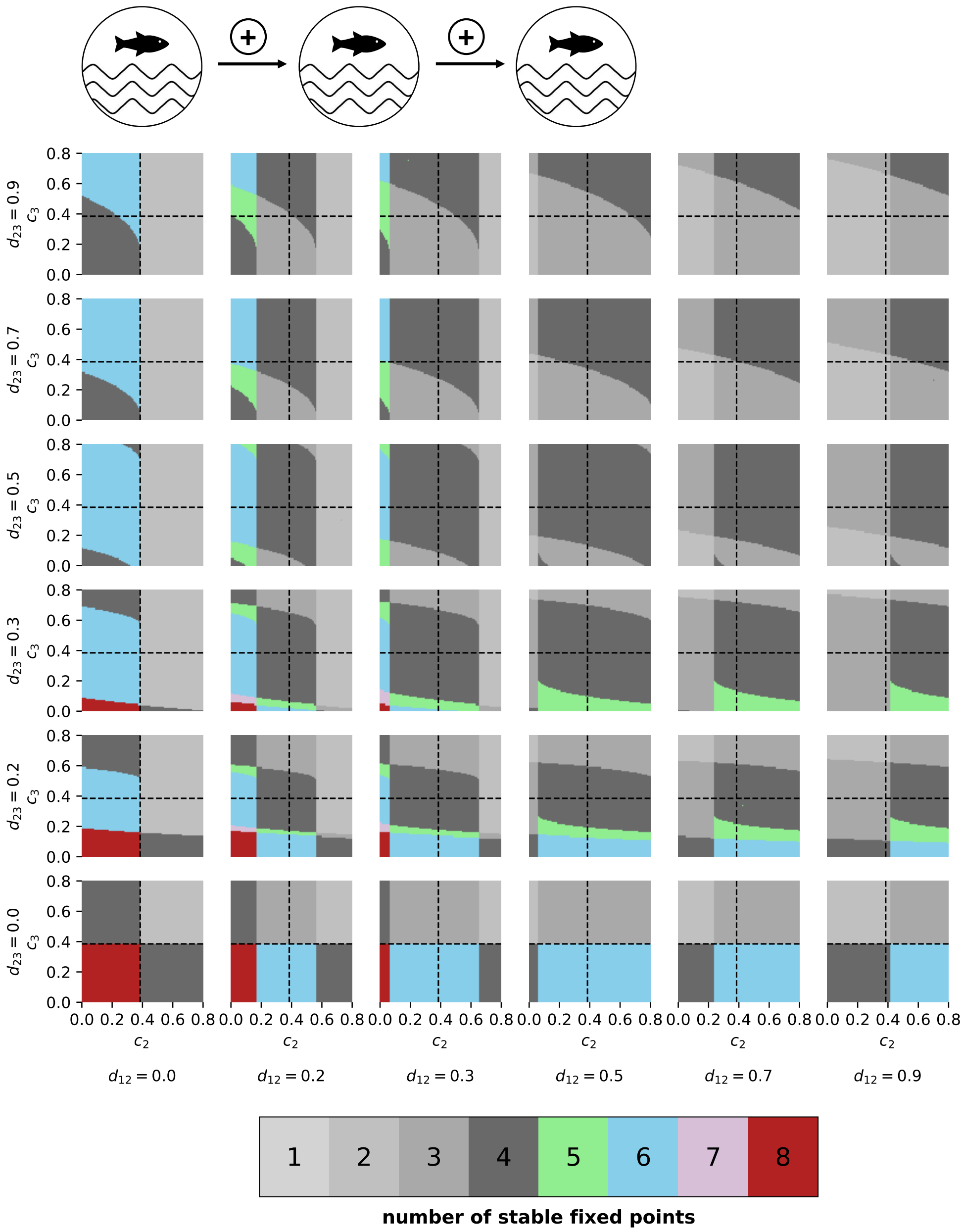


Figure 2: Number of stable fixed points of the system consisting of three unidirectionally coupled tipping elements for $c_1 = 0.2 < c_{1_{\text{crit}}}$ depending on the control parameters $c_2$ and $c_3$ and the coupling strengths $d_{12} \geq 0$ and $d_{23} \geq 0$ in a matrix of stability cards. A stability card shows the number of stable fixed points in the $(c_2, c_3)$–space for a specific coupling strength, where a certain number of stable fixed points is associated with a specific colour. Note that different areas in the control parameter space with the same colour have the same number of stable fixed point but they do not necessarily have the same phase portrait. The dashed lines represent the intrinsic tipping point of the respective subsystem. The position of a stability card in the matrix is determined by the coupling strength.

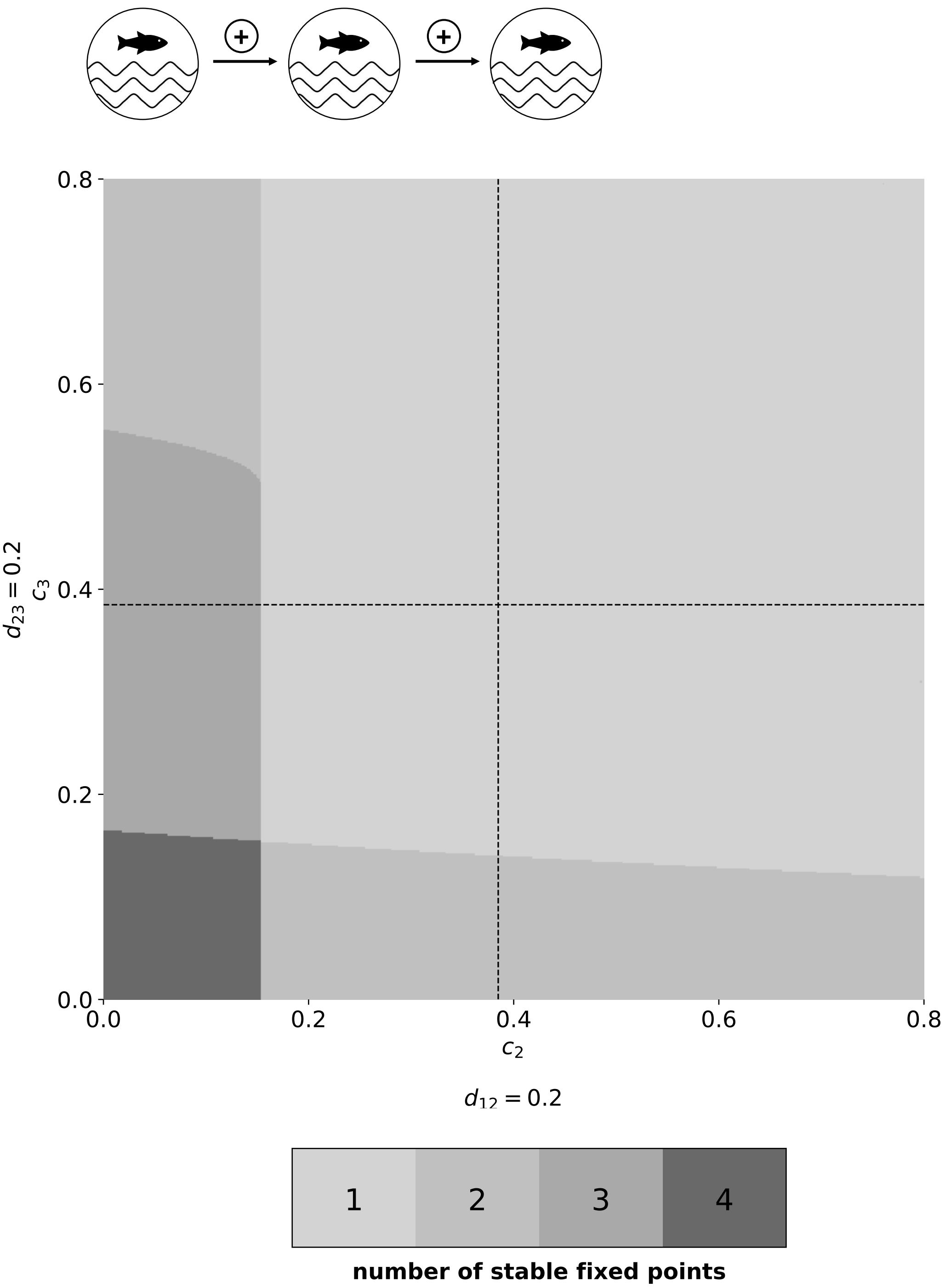


Figure 3: Number of stable fixed points of the system consisting of three unidirectionally coupled tipping elements for $c_1 = 0.4 > c_{1_{\text{crit}}}$ with $d_{12} = d_{23} = 0.2 > 0$ depending on the control parameters $c_2$ and $c_3$. The dashed lines represent the intrinsic tipping point of the respective subsystem.

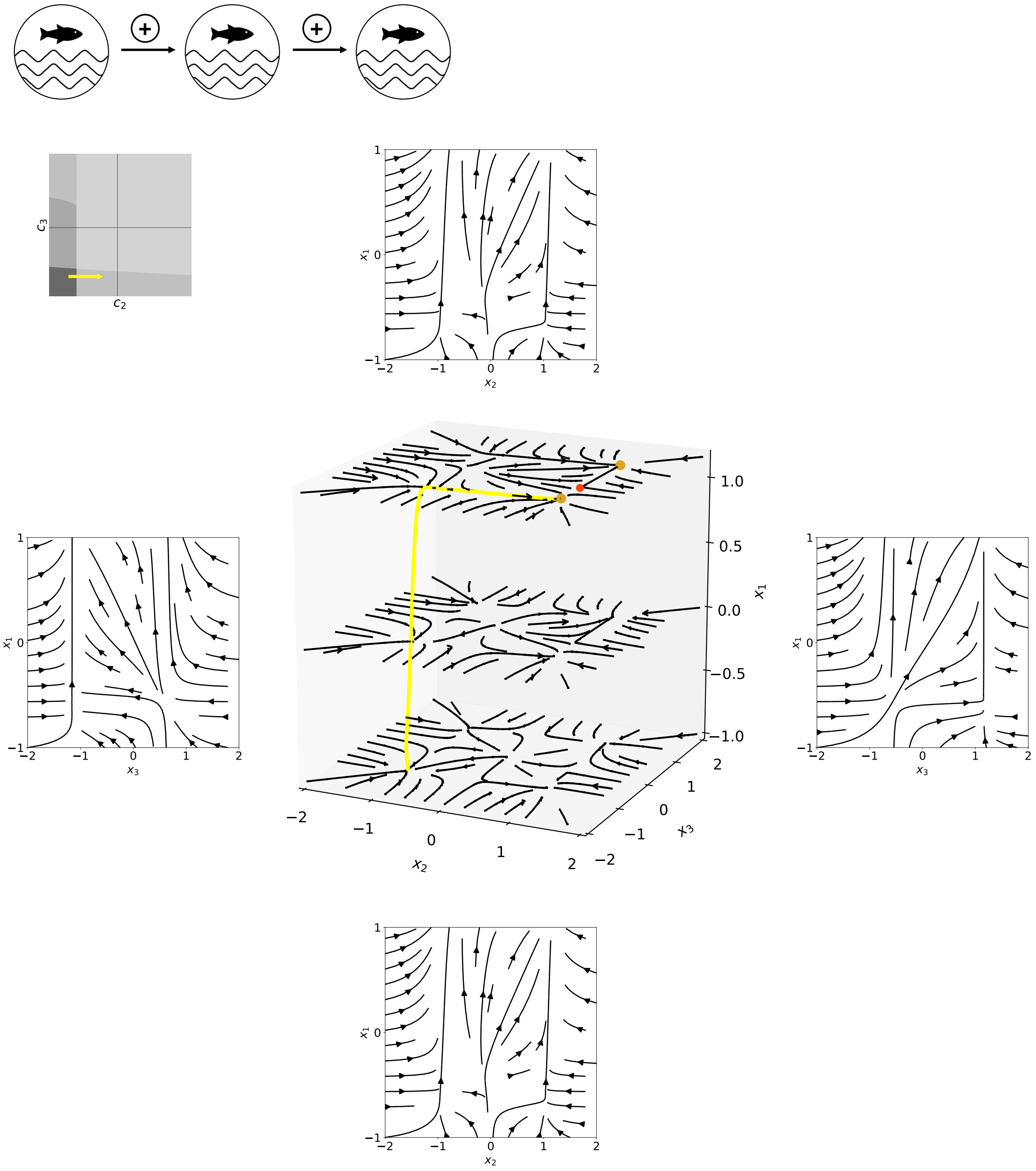

Figure 4: Facilitated tipping in a system of three unidirectionally coupled tipping elements for an increase of the control parameter $c_2 << c_{2_{\text{crit}}}$ (as indicated by the yellow arrow in the stability card, upper left panel). The central cube shows the flow in the $(x_2, x_3)$–space (in black) as part of the three–dimensional phase space and the remaining stable (in orange) as well as unstable (in red) fixed points for $c_1 = 0.4 > c_{1_{\text{crit}}}$, $c_2 = 0.2$ and $c_3 = 0.0$ with $d_{12} = 0.2 > 0$ and $d_{23} = 0.2 > 0$. An exemplary case of facilitated tipping in subsystem $X_2$, where the preceding subsystem $X_1$ gets to the alternative state for $c_1 > c_{1_{\text{crit}}}$, is highlighted by the yellow trajectory. Two–dimensional plots arranged around the central cube show the flow in the $(x_2, x_1)$– and $(x_3, x_1)$–space corresponding to the lateral surfaces of the cube.

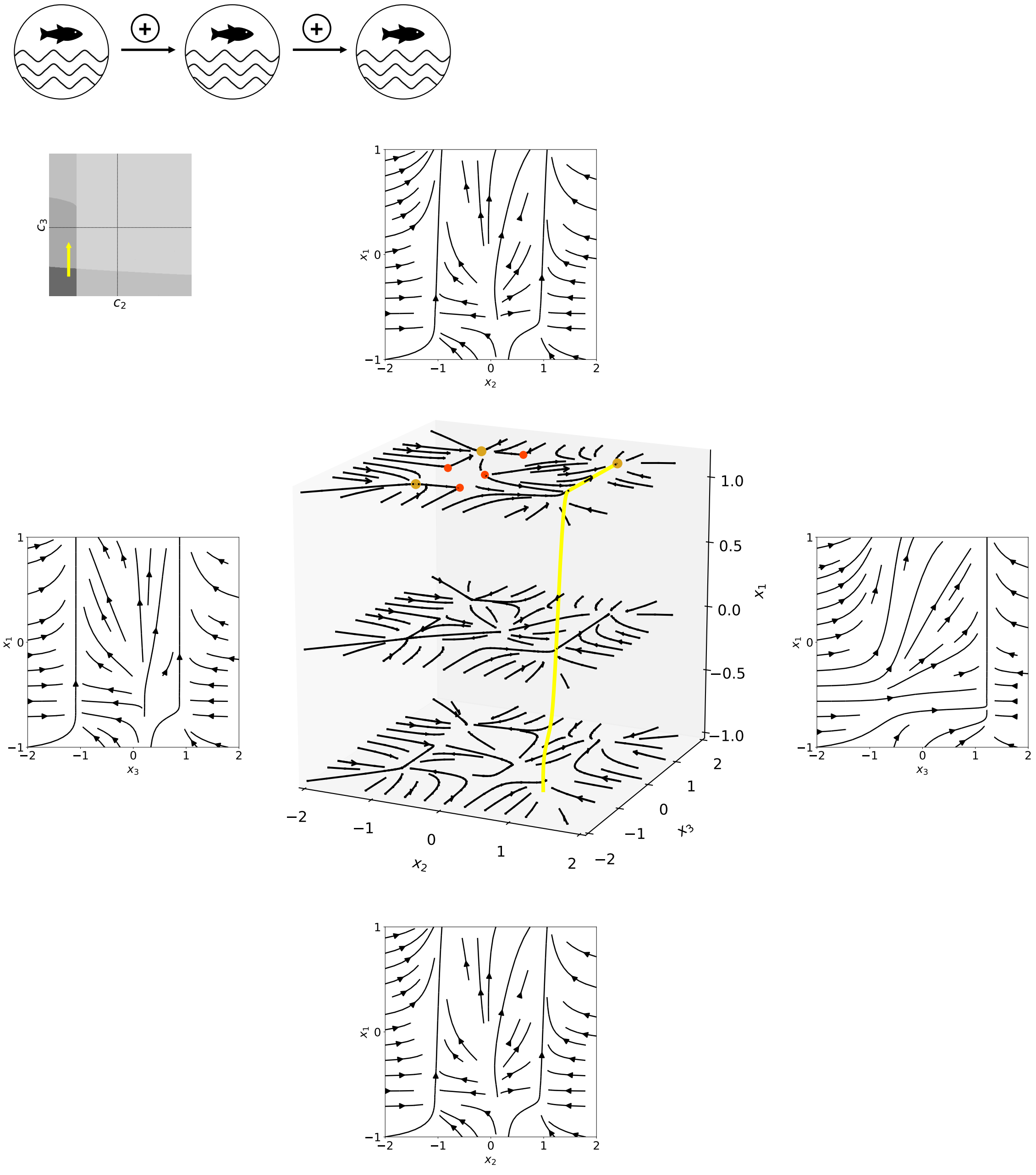

Figure 5: Facilitated tipping in a system of three unidirectionally coupled tipping elements for an increase of the control parameter $c_3 << c_{3_{\mathrm{crit}}}$ (as indicated by the yellow arrow in the stability card, upper left panel). The central cube shows the flow in the $(x_2, x_3)$–space (in black) as part of the three–dimensional phase space and the remaining stable (in orange) as well as unstable (in red) fixed points for $c_1 = 0.4 > c_{1_{\mathrm{crit}}}$, $c_2 = 0.0$ and $c_3 = 0.2$ with $d_{12} = 0.2 > 0$ and $d_{23} = 0.2 > 0$. An exemplary case of facilitated tipping in subsystem $X_3$, where the preceding subsystem $X_2$ occupies the alternative state, is highlighted by the yellow trajectory. Two–dimensional plots arranged around the central cube show the flow in the $(x_2, x_1)$– and $(x_3, x_1)$–space corresponding to the lateral surfaces of the cube.
